# COVID-19 Epidemic in Argentina: Worsening of Behavioral Symptoms in Elderly Subjects With Dementia Living in the Community

**DOI:** 10.3389/fpsyt.2020.00866

**Published:** 2020-08-28

**Authors:** Gabriela Cohen, María Julieta Russo, Jorge A. Campos, Ricardo F. Allegri

**Affiliations:** Memory and Ageing Center, Department of Cognitive Neurology, Neuropsychiatry and Neuropsychology, Fleni. Fundación para el estudio de enfermedades neurológicas de la infancia, Buenos Aires, Argentina

**Keywords:** dementia, behavioral symptoms, COVID-19 epidemic, quarantine, elderly

## Abstract

In Argentina, the quality of care that elderly subjects with dementia living in the community received has been deeply affected by COVID-19 epidemic. Our objective was to study to what extend mandatory quarantine imposed due to COVID-19 had affected behavioral symptoms in subjects with dementia after the first 8 weeks of quarantine. We invited family members to participate in a questionnaire survey. The sample consisted of family caregivers (n = 119) of persons with AD or related dementia living at home. We designed a visual analog scale to test the level of the burden of care of family members. Items inquired in the survey included type and setting (home or day care center) of rehabilitation services (physical/occupational/cognitive rehabilitation) and change in psychotropic medication and in behavioral symptoms that subjects with dementia experienced before and during the epidemic. Characteristics of people with dementia and their caregivers were analyzed with descriptive statistics using the chi-square tests, p < 0.01 was considered significant. Results: The sample included older adults with dementia. Mean age: 81.16 (±7.03), 35% of the subjects had more than 85 years of age. Diagnosess were 67% Alzheimer´s dementia and 26% mixed Alzheimer´s disease (AD). Stages were 34.5% mild cases, 32% intermediate stage, and 33% severe cases as per Clinical dementia Rating score. In 67% of the sample, a family member was the main caregiver. Important findings were increased anxiety (43% of the sample), insomnia (28% of the subjects), depression (29%), worsening gait disturbance (41%), and increase use of psychotropics to control behavioral symptoms. When we compared the frequency of behavioral symptoms within each dementia group category, we found that anxiety, depression, and insomnia were more prevalent in subjects with mild dementia compared to subjects with severe dementia. We analyzed the type and pattern of use of rehabilitation services before and during the isolation period, and we observed that, as a rule, rehabilitation services had been discontinued in most subjects due to the quarantine. We concluded from our analysis that during COVID-19 epidemic there was a deterioration of behavioral symptoms in our population of elderly dementia subjects living in the community. Perhaps, our findings are related to a combination of social isolation, lack of outpatient rehabilitation services, and increased stress of family caregivers. It is necessary to develop a plan of action to help dementia subjects deal with the increased stress that this epidemic imposed on them.

## Introduction

The World Health Organization declared the COVID-19 epidemic on March 11 (1). A few days later, and with the experience of how the epidemic was affecting countries in Europe and Asia, the Argentinian Government issued an executive order implementing a complete lockdown and isolation of travelers returning from the affected countries (2). Non-essential business was closed, and people were asked to avoid unnecessary travel to maintain social distance and to limit family visits to elderly subjects (2). At the time of writing this communication, quarantine in Buenos Aires had lasted 101 days, the number of deceased people in the entire country had reached 1,245, and the number of infected persons is 59,933 (3). Governments through the world were challenged to deal with both the direct impact of the disease on the health system and the economic, financial, and social consequences of the epidemic. Worldwide health authorities also need to design models of care of chronic conditions not related to COVID-19 in times of this epidemic (3).

Alzheimer`s disease (AD) and related disorders subjects are especially vulnerable to the effects of COVID-19 disease and the imposed quarantine (4, 5). Based on frequent comorbidities and older age, they might be at higher risk for severe illness from COVID-19 (5–7). For example, in Italy, dementia was one of the most frequent comorbidities present in 12% of the deceased COVID-19 patients (8). On the other hand, dementia subjects are also extremely vulnerable to the effects of enforced quarantine (9). They depend strongly on community and social support systems for survival due to the dependence on activities of daily living (10). They may not learn properly the use personal protection elements, such as wearing facial masks, washing hands, and keeping social distance, and may forget to avoid leaving their home unnecessarily. They are probably be less flexible on coping with changing situations and during crisis, and they rely more on family members. However, during this epidemic, family members are trying to limit contact with elderly dementia subjects to decrease as much as possible the risk of coronavirus transmission. While during this epidemic virtual technology is playing a central role in preventing isolation in the general population, this vital resource is sometimes difficult to utilize for dementia subjects due to their difficulties to learn the use of this technology (9–12).

As suggested by the Alzheimer`s International Society, support for subjects living with dementia and their caregivers is mandatory (11). Access to care for family members and dementia subjects in order to deal with a new situation is critical (8) and mitigation strategies to reduce the immediate and long-term impact of this health crisis are needed (4). For example, in Australia, the Health Department quickly realized the need for improved access to mental healthcare services for older people during COVID-19 times (13). While during this epidemic virtual technology is playing a central role in preventing isolation, this vital resource is difficult to utilize on dementia subjects due to their difficulties to learn the use of this technology (9–12).

To prepare a rational plan to mitigate the effects of the epidemic, it is necessary first to identify in our setting the most problematic situations that AD and related dementia subjects are facing. It is also important to determine if enforced isolation imposed specific issues related to the severity of the cognitive disease. We know that AD and related dementia neuropsychiatric symptoms, such as anxiety, depression, sleep difficulties, among others, are extremely frequent with a prevalence ranging from 60 to 80% and usually imposed more troubling on caregivers than the cognitive symptoms (14, 15). Sleep disturbances are reported in at least 30% of subjects with AD. Multifactorial contributors are depression, anxiety, sedentarism, and adverse reactions from medications (17). Standard nonpharmacologic proven strategies to improve these disrupting symptoms and commonly used by caregivers are maintaining a structured routine, reassuring responses, physical exercise, sleep hygiene, and distraction. Most of these strategies are difficult, almost impossible, to implement during the quarantine (16).

Our objective was to measure in our setting the impact of COVID-19 epidemic on the well-being and behavioral symptoms of subjects at different stages of dementia living in the community after the first 8 weeks of enforced isolation.

## Materials and Methods

Family members of patients of the Aging and Memory Center of FLENI with AD and related disorders were invited to participate in our survey. Two physicians (MJR and GC) of the Aging and Memory Center provided information about study´s aim and distributed the questionnaire survey. Participation was voluntary and anonymous. The survey sample consisted of family caregivers (n = 119) of persons with AD or related dementia living at home.

### Survey

The survey had two main sections. The first one included demographics of family members, paid caregivers, and dementia subjects, and the other was composed of questions regarding the challenges of care and management that subjects and relatives experienced during the first 8 weeks of the coronavirus quarantine in our setting. The survey was not intended to replace a medical office visit or to make a clinical diagnosis, and validated tests were not used. Our idea was to study with easy and quick to answer questions the psychological issues that might had occurred during quarantine. A series of questions were designed specifically to screen the onset or worsening of behavioral symptoms (anxiety, insomnia, and depression) or gait disturbances during the quarantine. We specifically asked caregivers the following two questions for each symptom inquired: “Did your relative with dementia experience anxiety before the epidemic?” and “Do your relative with dementia experience anxiety during the epidemic?” In order to study if there was a change in the prescription of psychotropics during quarantine, we asked caregivers specifically the following questions. “During quarantine your relative needed the dose of the following medications to be increased or to be started?” For each of the following medications, we asked one specific question. The list included: antipsychotics (quetiapine/risperidone/olanzapine), anxiolytics (clonazepam, alprazolam, diazepam), non-benzodiazepines hypnotics (zolpidem), and antidepressants (citalopram, escitalopram, sertraline, venlafaxine, fluoxetine, paroxetine, and trazodone). We did not record the exact dose of the psychotropics, but the reported change in the dose of the prescriptions.

A series of questions were made to assess the type and setting of rehabilitation services that subjects where receiving before the epidemic. We inquired specifically if subjects did physical/occupational and cognitive rehabilitation and if it was home based/at a day care center or specialized outpatient center. We then asked if rehabilitation services had been discontinued during the epidemic. We also asked if family members continued or discontinued visiting subjects during the quarantine.

### Patients

Patients were seen and studied extensively by a doctor specialized in memory disorders before COVID-19 epidemic. Clinical diagnosis of cognitive disorder syndromes was made based on a detailed workup of history taking, medication review, physical examination, neuroimaging, and neuropsychological tests. Disease severity was based on Clinical Dementia Ration (CDR) (18) score and functionality scales. All subjects had a longitudinal follow up in the memory disorder clinic. Due to the unique feature of isolation and quarantine in long term care facilities, we decided to include in our sample only subjects living in the community and excluded those living at long-term care settings.

### Family and Paid Caregivers

We designed a visual analog scale to study the burden of care that family members or paid caregivers experienced before and during the epidemic. The question was: “How much stress from 1 (low) to 3 (severe) do you feel by taking care of your family member with dementia before the quarantine and during quarantine?” We assigned 1 point for low, 2 points for intermediate, and 3 points for severe burden of care. Our intention was to measure the amount of burnout that a family caregiver feels ranges across a continuum from none to an extreme amount of stress. Based on the obtained score, the results were transformed into three categories: low, medium, and high. The next step was to understand the main concerns that family members were dealing with during quarantine in relation to the care of subjects with dementia. We created a list of six different hypothetical situations and asked family members to select the main concern from that list.

### Ethics

This study was presented and approved by the Medical Ethics Committee of our center. The participation of this survey was voluntary, and confidentiality of the dyad patient-family member was preserved through all research stages and after. A letter was mailed together with the questionnaire inviting family members to participate in the survey and informing them of the purpose of the research study.

### Statistical Analysis

Statistical analyses were made using IBM SPSS 21 software package. The characteristics of people with AD or related dementia and their caregivers were analyzed with descriptive statistics (percentages and means ± standard deviations). Chi-square tests, with p < 0.01 were used to test differences between family caregivers of persons in the mild stage and in severe stages of dementia based on CDR (18) score. Behavioral symptoms and covariates were analyzed with Spearman’s rank-order correlations. The level of burden of the family caregiver before and during the COVID-19 epidemic was analyzed with paired-samples t tests. To overcome some of the limitations imposed by conventional pretest-posttest self-report measures, the retrospective pretest-posttest design was utilized. We selected this method since it has been shown to reduce response-shift bias, p is convenient to implement and provides comparison data in the absence of “pre” data.

## Results

Our work is based on the data of a questionnaire survey collected during the month of May of 2020, after approximately 8 weeks of complete lockdown due to quarantine in Argentina.

Demographics and clinical characteristics of 119 subjects with AD and related dementia and their family members are shown in **Table 1**.

**Table 1:** Characteristics of participating caregivers and persons with Alzheimer’s disease or related dementia.

Variables	
**Part I: Persons with dementia**	
**Age, mean ± SD**	81.16 ± 7.03
<65 years old, n (%)	2 (1.7)
65–85 years old, n (%)	85 (71.4)
≥85 years old, n (%)	32 (26.9)
**Gender, male, n (%)**	42 (35.3)
**Education, mean ± SD**	13.26 ± 4.68
**Diagnosis, n (%)**	
AD	80 (67.2)
Mixed AD	26 (21.8)
Vascular dementia	7 (5.9)
Others	2 (1.7)
**CDR, mean ± SD**	1.99 ± 0.83
CDR 1, n (%)	40 (34.5)
CDR 2, n (%)	37 (31.9)
CDR 3, n (%)	39 (33.6)
**Increased or onset of COVID-19-related anxiety, n (%)**	50 (42)
**Increased or onset of COVID-19-related insomnia, n (%)**	34 (28.6)
**Increased or onset of COVID-19-related depression, n (%)**	35 (29.4)
**Increased gait problems during the COVID-19 pandemic, n (%)**	49 (41.2)
**Increased or onset of COVID-19-related antipsychotics prescription, n (%)**	24 (20.2)
**Increased or onset of COVID-19-related benzodiazepines prescription, n (%)**	18 (15.1)
**Increased or onset of COVID-19-related hypnotics prescription, n (%)**	8 (6.7)
**Increased or onset of COVID-19-related antidepressants prescription, n (%)**	12 (10.1)
**Physical therapy, n (%)**	
At home	47 (39.5)
Specialized centers or Senior Day Care Center	24 (20.2)
No therapy	48 (40.3)
**Occupational therapy, n (%)**	
At home	18 (15.1)
Specialized centers or Senior Day Care Center	12 (10.1)
No therapy	89 (74.8)
**Cognitive Rehabilitation, n (%)**	
At home	21 (17.6)
Specialized centers or Senior Day Care Center	29 (24.4)
No therapy	69 (58)
**Discontinued physical therapy during the COVID-19 pandemic, n (%)**	47/61 (76.9)
**Discontinued occupational therapy during the COVID-19 pandemic, n (%)**	21 (91.3)
**Discontinued cognitive rehabilitation during the COVID-19 pandemic, n (%)**	31/40 (77.5)
**Part II: Family caregivers**	
**Age, mean ± SD**	58.61 ± 13.60
<45 years old, n (%)	17 (14.9)
45–65 years old, n (%)	64 (56.1)
65–85 years old, n (%)	30 (26.3)
≥85 years old, n (%)	3 (2.6)
**Gender, male, n (%)**	32 (28.1)
**Education, mean ± SD**	17.04 ± 5.15
**Level of burden of the family caregiver prior to the pandemic, mean ± SD**	1.69 ± 0.67
Low burden, n (%)	51 (42.9)
Medium burden, n (%)	54 (45.4)
High burden, n (%)	14 (11.8)
**Level of burden of the family caregiver due to COVID-19, mean ± SD**	2.27 ± 0.72
Low burden, n (%)	19 (16)
Medium burden, n (%)	49 (41.2)
High burden, n (%)	51 (42.9)
**Discontinued visit to a family member with dementia during the COVID-19 pandemic, n (%)**	41 (34.5)
**Discontinued paid caregiver with dementia during the COVID-19 pandemic, n (%)**	27 (23.7)
**Questions about supporting someone with dementia during the coronavirus outbreak, n (%)**	
I’m concerned with how to handle disruptive behaviors while we are quarantined at home	32 (31.1)
I’m not sure how I explain the situation to a person with dementia?	11 (10.7)
I’m worried that my relative with dementia may worse during COVID-19 quarantine.	9 (8.7)
I’m worried that the professional caregivers who come in to help us might not be able to come.	13 (12.6)
I need to go outside to pick up supplies for my relative with dementia but I am worried that I might catch the virus	27 (26.2)
I’m concerned that the caregiver is exhausted by the quarantine	11 (10.7)
**Part III: Paid caregivers**	
**Paid caregiver, n (%)**	40 (33.6)
**Level of burden of the paid caregiver prior to the pandemic, mean ± SD**	1.35 ± 0.57
Low burden, n (%)	72 (69.9)
Medium burden, n (%)	26 (25.2)
High burden, n (%)	5 (4.9)
**Level of burden of the paid caregiver due to COVID-19, mean ± SD**	1.55 ± 0.75
Low burden, n (%)	62 (60.2)
Medium burden, n (%)	25 (24.3)
High burden, n (%)	16 (15.5)

This table represents valid percentage of responses on the questionnaire survey specifically designed for this study. The bold represents p < 0.05.

CDR, clinical dementia rating; AD, Alzheimer´s disease.

### Characteristics of Subjects With Dementia

Baseline demographics were the following: Mean age of subjects with dementia was 81.16 ± 7.03 years (a third of the sample belonged to the eldest-old group of more than 85 years of age), approximately a third were male, and mean number of years of education was 13.26 ± 4.68. The most frequent diagnosis was AD, followed by mixed AD, and then by vascular dementia. The distribution of the staging of dementia was the following: 34% of the sample had mild dementia (CDR 1), 32% had moderate dementia, and 34% had severe dementia with a CDR score of 3.

Our main result was the report by family members of new onset or exacerbation of pre-existing behavioral symptoms in 60.5% of subjects with dementia during the epidemic. Symptoms of anxiety, depression, and sleep disorders were reported in 33, 12.8, and 14.7% of the sample, respectively. Increasing gait difficulties was reported in 40% of the sample. New onset of behavioral symptoms or exacerbation of pre-existing behavioral symptoms had a positive correlation with patient age and with the presence of anxiety reported before the epidemic (r = 0.228, p = 0.017 and r = 0.290, p = 0.002, Spearman, respectively) and a negative correlation with the global CDR score (r = -0.289, p = 0.002, Spearman) and with the following domains of CDR: memory (r = -0.202, p = 0.035, Spearman), community affairs (r = -0.236, p = 0.013, Spearman), and home and hobbies (r = -0.216, p = 0.024, Spearman).

Family members reported an overall increased use of psychotropic medication during the epidemic with the following distribution: 20% increased for antipsychotics, 15% for benzodiazepines, 6% for hypnotics, and 10% for antidepressants.

**Figure 1:**
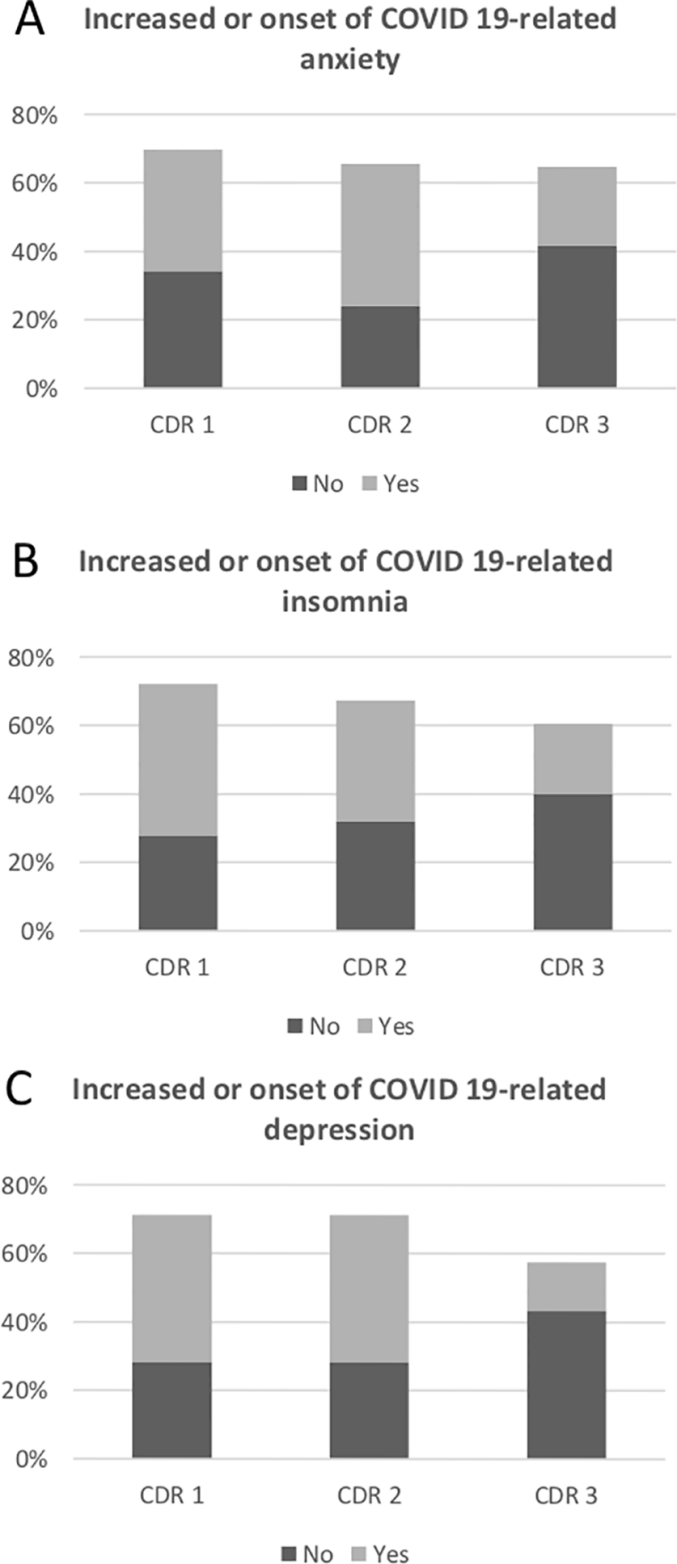
This figure detailing valid percentage of responses on the questionnaire survey specifically designed for this study. **(A)** Percentage of patients with increased or onset of anxiety during quarantine. **(B)** Percentage of patients with increased or onset of insomnia during quarantine. **(C)** Percentage of patients with increased or onset of depression during quarantine.

In **Table 2**, we compared data according to the stages of severity of dementia. We found significant differences in increased behavioral symptoms of anxiety, insomnia, and depression in subjects with mild dementia compared to subjects with a more advanced stage of dementia. These results were showed in **Figure 1**. For psychotropics, we observed a non-significant trend in increased prescription in the mild dementia group (**Table 2**). Walking difficulties didn´t differ significantly according to the disease severity.

**Table 2:** Characteristics of participating caregivers and persons with Alzheimer’s disease or related dementia according to Global Clinical Dementia Rating score.

Variables	CDR			T/χ2	*p*
	1	2	3		
**Part I: Persons with dementia**					
**Age**				3.861	0.425
<65 years old	50.00%	0.00%	50.00%		
65–85 years old	38.10%	28.60%	33.30%		
≥85 years old	23.30%	43.30%	33.30%		
**Gender, male**	24.40%	31.70%	43.90%	3.868	0.145
**Education**	13.3 ± 4.65	12.06 ± 4.82	14.53 ± 4.43	2.63	0.077
**Diagnosis**				7.866	0.447
AD	31.30%	32.50%	36.30%		
Mixed AD	42.30%	34.60%	23.10%		
Vascular dementia	42.90%	28.60%	28.60%		
Others	0.00%	0.00%	100.00%		
**Increased or onset of COVID-19-related anxiety**	35.40%	41.70%	22.90%	5.733	**0**.**05**
**Increased or onset of COVID-19-related insomnia**	44.10%	35.30%	20.60%	4.37	**0**.**037**
**Increased or onset of COVID-19-related depression**	42.90%	42.90%	14.30%	0.886	**0**.**012**
**Increased gait problems during the COVID-19 pandemic**	38.80%	32.70%	28.60%	0.423	0.809
**Increased or onset of COVID-19-related antipsychotics prescription**	33.30%	33.30%	33.30%	0.098	0.952
**Increased or onset of COVID-19-related benzodiazepines prescription**	27.80%	33.30%	38.90%	0.796	0.672
**Increased or onset of COVID-19-related hypnotics prescription**	12.50%	50.00%	37.50%	2.021	0.364
**Increased or onset of COVID-19-related antidepressants prescription**	41.70%	33.30%	25.00%	0.338	0.845
**Physical therapy**				13.003	**0**.**011**
At home	19.60%	30.40%	50.00%		
Specialized centers or Senior Day Care Center	33.30%	37.50%	29.20%		
No therapy	50.00%	30.40%	19.60%		
**Occupational therapy**				5.595	0.232
At home	11.10%	38.90%	50.00%		
Specialized centers or Senior Day Care Center	41.70%	33.30%	25.00%		
No therapy	38.40%	30.20%	31.40%		
**Cognitive Rehabilitation**				7.251	0.123
At home	28.60%	33.30%	38.10%		
Specialized centers or Senior Day Care Center	48.30%	37.90%	13.80%		
No therapy	30.30%	28.80%	40.90%		
**Discontinued physical therapy during the COVID-19 pandemic**	25.50%	36.20%	38.30%	2.802	0.246
**Discontinued occupational therapy during the COVID-19 pandemic**	14.30%	42.90%	42.90%	4.65	0.098
**Discontinued cognitive rehabilitation during the COVID-19 pandemic**	29.00%	41.90%	29.00%	1.966	0.374
**Part II: Family caregivers**					
**Age**				4.714	0.581
<45 years old	41.20%	17.60%	41.20%		
45–65 years old	32.80%	36.10%	31.10%		
65–85 years old	26.70%	36.70%	36.70%		
≥85 years old	66.70%	0.00%	33.30%		
**Gender**	31.30%	31.30%	37.50%	0.218	0.897
**Education**	15.95 ± 4.94	18.39 ± 6.28	16.87 ± 4.10	2.08	0.13
**Level of burden of the family caregiver prior to the pandemic**				5.377	0.251
Low burden	35.30%	31.40%	33.30%		
Medium burden	40.40%	30.80%	28.80%		
High burden	7.70%	38.50%	53.80%		
**Level of burden of the family caregiver due to COVID-19**				4,538	0.338
Low burden	47.40%	31.60%	21.10%		
Medium burden	33.30%	25.00%	41.70%		
High burden	30.60%	38.80%	30.60%		
**Discontinued visit to a family member with dementia during the COVID-19 pandemic**	0.268	0.317	0.415	2.238	0.327
**Discontinued paid caregiver with dementia during the COVID-19 pandemic**	0.259	0.333	0.407	1.037	0.595
**Questions about supporting someone with dementia during the coronavirus outbreak**					
I’m worried that the professional caregivers who come in to help us might not be able to come.	0.077	0.462	0.462	3.645	**0**.**05**
I need to go outside to pick up supplies for my relative with dementia but I am worried that I might catch the virus	0.519	0.296	0.185	5.731	**0**.**02**
**Part III: Paid Caregivers**					
**Paid caregiver**	22.50%	27.50%	50.00%	7.784	**0**.**02**
**Level of burden of the paid caregiver prior to the pandemic, mean ± SD**				7.464	0.113
Low burden	33.30%	36.20%	30.40%		
Medium burden	26.90%	19.20%	53.80%		
High burden	0.00%	60.00%	40.00%		
**Level of burden of the paid caregiver due to COV6ID-19, mean ± SD**				3.588	0.465
Low burden	35.00%	35.00%	30.00%		
Medium burden	20.80%	29.20%	50.00%		
High burden	25.00%	31.30%	43.80%		

This table represents valid percentage of responses on the questionnaire survey specifically designed for this study. The bold represents p < 0.05.

CDR, Clinical Dementia Rating; AD, Alzheimer´s disease.

Before the epidemic, the most commonly prescribed type of rehabilitation was physical therapy (60% of the sample), followed by cognitive rehabilitation in 42%, and by occupational therapy in a lower percentage (25%) (**Table 1**). As expected, subjects with more severe dementia received home-based physical therapy (**Table 2**). There was a high rate of discontinuation of rehabilitation during the epidemic: 76% discontinued physical therapy, 91% occupational therapy, and 77% cognitive rehabilitation. There was no statistical difference in the rate of discontinuation based on the severity of dementia.

### Characteristics of Family Members and Paid Caregivers

In **Table 1**, we showed the demographic characteristics of family members and paid caregivers. The mean age of the family members was 58.61 ± 13.60 years, 26% were older than 65 years of age, and 2% older than 85%. As expected, most family members caring for patients were female. The mean education of the family caregivers was 17.04 ± 5.15 years. Another aspect of care that we wanted to study was the discontinuation of family visits during the COVID-19 epidemic. We found that most family members continued to visit their loved ones during quarantine with a discontinuation rate of only 34%.

Overall, we observed an increased burden of care of family members during the epidemic, independently of the dementia severity. 12% of the family members felt that the burden of care was severe before the epidemic, and this number increased to 42% during the epidemic. Thus, there was a significant difference in the burnout scores before (M = 1.69, SD = 0.67) and during (M = 2.27, SD = 0.72) the COVID-19 epidemic; t = -8,657, p < 0.001. When we analyzed the reasons for the increased family burnout, we found interesting differences. Relatives of severe dementia subjects were mainly concerned of the possibility of a sick leave of paid caregivers, whereas relatives of subjects with mild dementia were mainly concerned of the risk of COVID-19 transmission when assisting subjects in instrumental activities of daily living.

Before the epidemic, 40% of the sample received care from a paid caregiver. More severe cases tended to receive care from a paid caregiver compared to milder cases. During the epidemic, only 23% of the sample discontinued this service.

## Discussion

This is a report of a survey of the well-being and aspects of care of 119 subjects living with dementia in the community and their family caregivers in Argentina during the initial 8 weeks of mandatory isolation due to COVID-19 epidemic.

Our sample was composed of elderly subjects with dementia, a third of those belonged to the very old group of elderly patients, and a third of the sample were men. As expected to this age group, the most frequent diagnosis was AD and followed by mixed AD. The severity of the dementia was evenly distributed, a third had mild disease, a third had intermediate disease, and a third had severe disease.

Overall, we found worsening or new onset of behavioral symptoms of anxiety, depression, and insomnia during the enforced quarantine in subjects with dementia. There was a positive correlation of these symptoms with advanced age and with the presence of anxiety before this epidemic and a negative correlation with the global CDR (18) score, community affairs, and hobbies domain of CDR scale. Other findings were that most family members continued family visits during the epidemic, only a small proportion canceled caregiver paid services, and most rehabilitation services were discontinued during the epidemic.

Our findings are worrisome since behavioral and psychological symptoms associated to dementia are a main cause of deterioration of quality of life for patients and caregivers, institutionalization, disability, increased use of health resources and caregiver stress (15, 16). Longitudinal studies of dementia subjects showed that these symptoms are highly prevalent and persistent over time and can occur at any point in the clinical course of the cognitive process (19, 20). Non-pharmacologic management is consistently recommended in the literature to control these symptoms due, in part, of the modest efficacy and the potential of harm of pharmacologic therapy (16). Caregiver training, keeping the patient active with a structured personalized routine, taking the patient for a walk-in neighborhood are all well-proven strategies to deal with anxiety and agitation in patients with dementia (16). Unfortunately, during enforce isolation, some of these strategies were impossible to implement since Argentinian authorities recommended that high-risk subjects with comorbidities remain at home (2). Most forms of rehabilitation interventions had been cancelled. Home-based interventions were probably cancelled because of fear of letting a health care professional enter patient’s home and increasing the risk of spreading the epidemic. Outpatient rehabilitation services had been cancelled as a direct effect of quarantine to avoid unnecessary travel. Evidence from small trials in dementia showed that cognitive training and rehabilitation could improve cognition and decreased psychological symptoms. A recent review of reviews showed that exercise improved performance of daily activities in dementia (21). In our study, most interventions were suspended, and there is probably a relationship with the negative psychological issues found during quarantine and the cancellation of rehabilitation services.

Another main finding of our research was that neuropsychiatric symptoms during quarantine were more frequent in subjects with mild dementia than in advanced dementia cases. One possible explanation for this could be that comparatively, mild dementia subjects might have suffered more radical modification in their lifestyle habits during quarantine than subjects with severe dementia who usually are more homebound and less active.

Anxiety is reported in the literature to be strongly related to impairment of activities of daily living and dependence. In our sample, anxiety was the most frequently behavioral symptom experienced by dementia subjects during quarantine, and it was most frequently suffered by subjects with mild dementia. It is possible that, during quarantine, these subjects had more awareness of epidemic and risks of getting sick and that this knowledge induced more anxiety.

Sleep disturbances are frequent in AD patients and are related to age changes in sleep patterns, medication effects, comorbidity with anxiety, depression, and to the neurodegenerative disease by itself (18). Sleep disorders are disruptive to caregivers and increase the rate of institutionalization and caregiver burnout (22, 23). Strategies to improve sleep quality include sleep hygiene, physical activity during the day, and keeping a structured daily routine (22, 23). These strategies were all compromised during this lockdown period, and sleep difficulties were overall frequent, with even higher prevalence in subjects with mild dementia. Specially in this population, sedentary behavior during quarantine could had impacted on the quality of sleep of subjects with mild dementia

Psychotropic medication use increased during quarantine, independently of the dementia severity. Specifically use of antipsychotics, benzodiazepines, hypnotics, and antidepressants were more frequently prescribed. The first three medications are included in the Beer´s list of potentially inappropriate medication in elderly subjects with dementia (24). These medications have the potential for cognitive decline and increase the risk of falling and confusional state (24). Also, antipsychotics in this vulnerable group increased the risk of worse cardiovascular outcomes and are not currently recommended (25). This increased in use of potentially inappropriate medications in the elderly could cause in the future a deleterious effect on the health status of the subjects in our sample. A medication reconciliation plan once the quarantine ends with an active deprescribing strategy is one possible strategy to mitigate this increased risk.

Another main finding of our study is that there was a deterioration of the quality of walking during the quarantine. Gait impairment is frequent in dementia patients, especially in frail elderly subjects with advanced dementia, and is directly related to the risk of falling and quality of life of the subjects (26, 27). The performance of functional capacities depends on the ability to ambulate (27). Walking deterioration during quarantine is probably multifactorial, including discontinuation of physical and cognitive rehabilitation, deconditioning related to staying at home, and increased use of psychotropics as described above.

Probably, one of the most important learnings of this epidemic is the inclusion of technology for the evaluation and monitoring of our patients at a distance, even in older adults. While technology now is being used to socialize and give emotional support and guidance to caregivers, cognitive and physical exercise can be delivered *via* internet (28). It is true than some individuals may struggle to use this technology (29), by contrast, most caregivers usually can successfully use this resource. A recent published randomized trial of a specialized dementia care program delivered this way to the dyad patient-caregiver showed improved quality of life, decreased caregiver burn out and depression in those assigned to the active intervention (30).

Our study’s main limitations are the relatively small size of the sample and the lack of prospectively longitudinal follow-up. Another pitfall is the lack of use of validated instruments to measure caregiver burnout and behavioral and psychological symptoms. We will continue to follow this cohort of subjects to study the health consequences and the real impact after the isolation period, and we will continue our research using validated scales to measure these symptoms.

Our findings, in summary, showed the negative consequences of quarantine in this sample of elderly patients. Individuals with cognitive disorders are especially vulnerable during these times of isolation and epidemic, their care needs are not met, and social engagement is decreased. Caregivers and patients need more medical attention, support groups, and virtual modalities to deal with worsened behavioral symptoms, caregiver stress and burnout, walking abnormalities, and increased use of psychotropics. In general, most office consults had been cancelled, and caregiver have less contact and guidance with specialized medical teams than before COVID-19. Physical social distance required during the epidemic suspended interventions that subjects with dementia constantly need due to the chronic nature of cognitive decline. Family member’s awareness of the potential problems and a mitigation plan of action may help families deal with the negative impact of this natural crisis. Solutions will have to be creative, patient-centered, and flexible to deal with the new changing scenario. More medical counseling, guidance, and presence are needed urgently to help this population deal with new serious health challenges arose during the epidemic. Telehealth visits and telemedicine are a priority, and it must be implemented on a regular basis to provide frequent weekly medical counsel on specific new health issues related to this quarantine. Rehabilitation services also will have to adapt to the new scenario, with reduced occupancy of patients in the same area, among other strategies (31). It is necessary to urgently develop a plan of action to help dementia subjects and family members deal with the increased stress that his epidemic imposed on them.

## Data Availability Statement

The raw data supporting the conclusions of this article will be made available by the authors, without undue reservation.

## Ethics Statement

The studies involving human participants were reviewed and approved by FLENI. Ethics Committee. Written informed consent for participation was not required for this study in accordance with the national legislation and the institutional requirements.

## Author Contributions

The study was designed and conceived by GC, MR, JC, and RA. Recruitment of participants and data collection were undertaken by GC and MR. Statistical analysis was overseen by RA, and analysis was undertaken by GC and MR. The manuscript was prepared by GC and critically reviewed and approved by all authors.

## Conflict of Interest

The authors declare that the research was conducted in the absence of any commercial or financial relationships that could be construed as a potential conflict of interest.
